# Regulation of *Mycobacterium*-Specific Mononuclear Cell Responses by 25-Hydroxyvitamin D_3_

**DOI:** 10.1371/journal.pone.0021674

**Published:** 2011-06-28

**Authors:** Corwin D. Nelson, Brian J. Nonnecke, Timothy A. Reinhardt, W. Ray Waters, Donald C. Beitz, John D. Lippolis

**Affiliations:** 1 Ruminant Diseases and Immunology Research Unit, National Animal Disease Center, Agricultural Research Service, United States Department of Agriculture, Ames, Iowa, United States of America; 2 Bacterial Diseases of Livestock Research Unit, National Animal Disease Center, Agricultural Research Service, United States Department of Agriculture, Ames, Iowa, United States of America; 3 Department of Biochemistry, Biophysics, and Molecular Biology, Iowa State University, Ames, Iowa, United States of America; 4 Department of Animal Science, Iowa State University, Ames, Iowa, United States of America; McGill University, Canada

## Abstract

The active vitamin D metabolite, 1,25-dihydroxyvitamin D_3_ (1,25(OH)_2_D_3_), has been shown to be an important regulator of innate and adaptive immune function. In addition, synthesis of 1,25(OH)_2_D_3_ from 25-hydroxyvitamin D_3_ (25(OH)D_3_) by the enzyme 1α-hydroxylase in monocytes upon activation by TLR signaling has been found to regulate innate immune responses of monocytes in an intracrine fashion. In this study we wanted to determine what cells expressed 1α-hydroxylase in stimulated peripheral blood mononuclear cell (PBMC) cultures and if conversion of 25(OH)D_3_ to 1,25(OH)_2_D_3_ in PBMC cultures regulated antigen-specific immune responses. Initially, we found that stimulation of PBMCs from animals vaccinated with *Mycobacterium bovis* (*M. bovis*) BCG with purified protein derivative of *M. bovis* (*M. bovis* PPD) induced 1α-hydroxylase gene expression and that treatment with a physiological concentration of 25(OH)D_3_ down-regulated IFN-γ and IL-17F gene expression. Next, we stimulated PBMCs from *M. bovis* BCG-vaccinated and non-vaccinated cattle with *M. bovis* PPD and sorted them by FACS according to surface markers for monocytes/macrophages (CD14), B cells (IgM), and T cells (CD3). Sorting the PBMCs revealed that 1α-hydroxylase expression was induced in the monocytes and B cells, but not in the T cells. Furthermore, treatment of stimulated PBMCs with 25(OH)D_3_ down-regulated antigen-specific IFN-γ and IL-17F responses in the T cells, even though 1α-hydroxylase expression was not induced in the T cells. Based on evidence of no T cell 1α-hydroxylase we hypothesize that activated monocytes and B cells synthesize 1,25(OH)_2_D_3_ and that 1,25(OH)_2_D_3_ down-regulates antigen-specific expression of IFN-γ and IL-17F in T cells in a paracrine fashion.

## Introduction

Substantial evidence supports the notion that vitamin D insufficiency (serum 25(OH)D_3_ concentrations <32 ng/mL or 80 nM) results in inadequate immune function and thus increased risk for infectious and autoimmune diseases [1]. For instance, an inverse correlation exists between serum 25(OH)D_3_ and the risk for upper respiratory tract infections [2], tuberculosis [3], [4], and multiple sclerosis [5], [6]. Vitamin D supplementation also decreases the risk influenza A infection [7], decreases the relapse rate in multiple sclerosis patients [8], and enhances ex vivo immunity to *Mycobacteria tuberculosis* [9]. The actions of 1,25-dihydroxyvitamin D_3_ (1,25(OH)_2_D_3_; the active hormone) on innate and adaptive immunity and the ability of immune cells to synthesize 1,25(OH)_2_D_3_ [10] provides further evidence for a link between vitamin D status and immune function. Understanding the mechanisms of vitamin D signaling in the immune system, consequently, provides critical insight for the vitamin D requirements of the immune system.

The vitamin D hormone has been known for some time to regulate key responses of innate and adaptive immunity. The actions of 1,25(OH)_2_D_3_ on the immune system occur through the vitamin D receptor (VDR). The VDR is present in most populations of immune cells [11], [12], [13] and controls the expression of genes that have promoters with accessible vitamin D response elements [14], [15]. In human monocytes and macrophages, 1,25(OH)_2_D_3_ induces cathelicidin, CD14, defensin beta 4, and NOD2 gene expression [16], [17], [18], [19]. In contrast to human monocytes, 1,25(OH)_2_D_3_ enhances inducible nitric oxide synthase (iNOS) and RANTES/CCL5 gene expression in bovine monocytes [20]. In regards to adaptive immunity, 1,25(OH)_2_D_3_ is a potent suppressor of lymphocyte proliferation and this has been observed for humans, cattle, and mice [21], [22], [23], [24]. In addition, 1,25(OH)_2_D_3_ suppresses IFN-γ responses of T cells from humans, cattle and mice in vitro [25], [26], [27], [28], [29]. Recently, 1,25(OH)_2_D_3_ also was found to suppress IL-17A responses of human and mouse T cells [25], [30], [31]. In mouse models of autoimmune disease, 1,25(OH)_2_D_3_ suppresses Th1 and Th17-mediated inflammation [32], [33], [34], and T cell VDR expression is required for 1,25(OH)_2_D_3_-mediated inhibition of experimental autoimmune encephalomyelitis (EAE) [35]. Altogether, in vitro and in vivo evidence show that 1,25(OH)_2_D_3_ acts on immune cells to regulate both innate and adaptive immunity, and that the actions of 1,25(OH)_2_D_3_ on adaptive immunity are similar among humans, cattle and mice.

The metabolism of 1,25(OH)_2_D_3_ is critical for immune function because of the potent effects of 1,25(OH)_2_D_3_ on innate and adaptive immunity. The enzyme that synthesizes 1,25(OH)_2_D_3_ from 25-hydroxyvitamin D_3_ (25(OH)D_3_) is 1α-hydroxylase (1α-OHase) [36]. In the vitamin D endocrine system, 1α-OHase is expressed in the kidney and is tightly regulated in response to calcium homeostasis via the parathyroid hormone in order to control the circulating concentration of 1,25(OH)_2_D_3_ [37]. However, the circulating concentration of 1,25(OH)_2_D_3_ does not affect vitamin D-mediated immune responses [38], [39] and circulating 1,25(OH)_2_D_3_ does not increase when the immune system is activated [40]. Rather, monocytes and macrophages express 1α-OHase in response to toll-like receptor (TLR) signaling, and this has been shown for humans, cattle, and mice [20], [41], [42]. In addition, dendritic cells, B cells and T cells also have been found to express 1α-OHase to some degree upon activation [43], [44]. However, 1α-OHase is predominantly upregulated in the CD14^+^ cells (monocytes/macrophages) from the inflamed mammary gland during mastitis in cattle [45]. Consequently, induction of 1α-OHase in immune cells enables regulation of 1,25(OH)_2_D_3_ concentration at sites of inflammation and this localized regulation is evident from animal models of inflammation. In cattle, the gene for 24-hydroxylase, the vitamin D catabolic enzyme that is highly upregulated by 1,25(OH)_2_D_3_, is expressed much higher in inflamed mammary tissue than in healthy tissue or circulating immune cells during mastitis [45]. Also in cattle, 1,25(OH)_2_D_3_ accumulated in granulomas during tuberculosis [46]. Finally, the concentration of 1,25(OH)_2_D_3_ increased in the spinal cords of mice during EAE, but did not change in serum [47]. Therefore, the immune system has a mechanism to control 1,25(OH)_2_D_3_ concentration locally independent of the endocrine system.

Subsequently, local control of 1,25(OH)_2_D_3_ metabolism by the immune system has been shown to have a significant impact on innate immunity [48]. For example, synthesis of 1,25(OH)_2_D_3_ by 1α-OHase in human monocytes induces their expression of cathelicidin [41]. Similarly, synthesis of 1,25(OH)_2_D_3_ by 1α-OHase in bovine monocytes enhances their expression of iNOS and RANTES [20]. So, 1,25(OH)_2_D_3_ produced in monocytes acts in an intracrine fashion to regulate vitamin-responsive genes.

As for adaptive immunity, monocyte production of 1,25(OH)_2_D_3_ has been suggested to also regulate T cell responses in a paracrine fashion [48]. However, lymphocytes also may be a source of 1,25(OH)_2_D_3_ and regulation of antigen-specific immune responses of T cells by conversion of 25(OH)D_3_ to 1,25(OH)_2_D_3_ in either monocytes or lymphocytes has yet to be shown. Therefore, the objectives of this study were to evaluate 1α-OHase gene expression in PBMC cultures in response to antigen stimulation and determine the effects of 25(OH)D_3_ on innate and adaptive immune responses in PBMC cultures.

To accomplish the objectives of this study we use PBMCs from calves vaccinated with *Mycobacterium bovis* bacilli Calmette-Guerin (*M. bovis* BCG), which elicits strong IFN-γ and IL-17 responses to purified protein derivative (PPD) of *M. bovis* [49]. The calf immune system has been found to serve as a good model of the human immune system for the study of tuberculosis and *M. bovis* BCG vaccination [50], [51]. In addition, the concentration of 25(OH)D_3_ circulating in blood is similar between cattle and humans with typical concentrations ranging from 20 to 100 ng/mL in both species [52], [53], [54]. In cattle and humans symptoms of vitamin D toxicity is rarely observed with circulating 25(OH)D_3_ concentrations below 200 ng/mL [8], [55], [56]. Finally, as mentioned already, local control of 1,25(OH)_2_D_3_ synthesis by the immune system and 1,25(OH)_2_D_3_ –regulation of T cell responses is similar between cattle and humans. Therefore, the outcome of this study will provide insight on the mechanisms of vitamin D signaling in the human and bovine immune systems.

## Materials and Methods

### Animals

Twelve male Holstein calves that were approximately 5 months to 12 months of age were used for this study. At 14 d of age, 8 calves were vaccinated subcutaneously in the midcervical region with 10^7^ cfu of *M. bovis* BCG (Pasteur strain). *M. bovis* BCG was prepared for vaccination as previously described [57]. The remaining 4 calves were not vaccinated. The NADC animal care and use committee approved the care and treatment of animals used in this study (Animal Protocol #ARS-3982).

### Peripheral blood mononuclear cell cultures

Blood from the jugular vein was collected in 2× acid citrate dextrose. Blood was centrifuged and buffy coats were collected. Contaminating RBCs were removed by hypotonic lysis. PBMCs were isolated by density gradient centrifugation. PBMC were resuspended in RPMI 1640 (Sigma-Aldrich, St. Louis, MO) supplemented with 50 µg/ml gentamicin (Invitrogen, Carlsbad, CA). For gene expression assays, PBMCs were cultured at a concentration of 1.5×10^7^ cells/ml in 96-well (200 µl/well) or 6-well (2 ml/well) tissue culture plates for 24 h at 37°C in 5% CO_2_. For determination of nitric oxide and IFN-γ production, PBMCs were cultured at 1×10^6^ cells/mL in a 96-well plate (200 µl/well) for 24, 48, and 72 h.

LPS from *Serratia marcescens* (Sigma-Aldrich), pokeweed mitogen (PWM; Sigma-Aldrich) and purified protein derivative from *M. bovis* (*M. bovis* PPD) (Prionics, Zurich, Switzerland) were added at 100 ng/ml, 5 µg/ml, and 10 µg/ml, respectively, to PBMC cultures. The vitamin D metabolites, 25(OH)D_3_ and 1,25(OH)_2_D_3_, (Sigma-Aldrich) were diluted in 100% ethanol and added to fetal bovine serum (FBS; Hyclone, Waltham, MA) at 10× the final desired concentration. The concentrations of 25(OH)D_3_ and 1,25(OH)_2_D_3_ in ethanol were confirmed by UV spectroscopy. FBS with ethanol (vehicle) or the vitamin D metabolites was added to PBMC cultures to a final concentration of 10% FBS. The final concentration of ethanol did not exceed 0.04%.

### Cell sorting

PBMCs from 7 BCG-vaccinated and 4 non-vaccinated calves were cultured with 10 µg/ml *M. bovis* PPD and 0 or 100 ng/ml 25(OH)D_3_ for 24 h in 6 well plates. Cells were removed from the wells with cold PBS and scraping. Cells were labeled with anti-CD14 (CAM36A; mouse IgG1), anti-IgM (PIG45A; mouse IgG2b), or a cocktail of anti-CD3 (MM1A; mouse IgG1), anti-CD4 (CACT83B; mouse IgM), anti-CD8 (MAQ111A; mouse IgM) and anti-γδTCR (CACT61A; mouse IgM). Cells labeled with the cocktail of CD3, CD4,CD8, and γδTCR antibodies are simply referred to as CD3^+^ cells. All primary antibodies were purchased from VMRD, Pullman, WA. Cells were then labeled with anti-mouse IgG1-PE (Becton Dickinson, San Jose, CA), anti-mouse IgG2b-Cy5 (Southern Biotech, Birmingham, AL), or a combination of anti-mouse IgG1-PE and anti-mouse IgM-PE (Becton Dickinson) secondary antibodies. Labeled cells were separated based on fluorescence intensity using the BD FACSAria Cell Sorting System (BD Biosciences, San Jose, CA). Approximately 10^6^ cells of each sub-population with >95% purity were collected from each PBMC culture.

### Relative gene expression

RNA was isolated from PBMC using the RNeasy Mini Kit (Qiagen, Valencia, CA). RNA samples were reverse transcribed to cDNA in 20 µl reactions using the High Capacity Reverse Transcription Kit with RNase inhibitor and random primers (Applied Biosystems, Foster City, CA). The reverse transcription reactions were incubated for 2 h at 37°C followed by 5 s at 85°C and finally cooled to 4°C. The cDNA samples were diluted 1∶10 in water and stored at −20°C.

The amount of specific cDNA transcripts in each sample was determined using the 7300 Real-Time PCR System (Applied Biosystems). Each reaction contained 12.5 µl SYBR Green Master Mix (Applied Biosystems), 7.5 µl of cDNA sample, and 5 µl of 10 µ*M* forward and 10 µ*M* reverse primers. Reactions were incubated as follows: 95°C for 10 min followed by 40 cycles of 95°C for 15 s and 60°C for 60 s. Primer sets were designed with Primer3 (http://frodo.wi.mit.edu/primer3) [58] to span intron-exon boundaries and are listed in Table 1. Primers were purchased from Integrated DNA Technologies (Coralville, IA). The efficiency of each primer set was determined as previously described [20] and fit the criteria required for quantification by real-time PCR [59]. The specificity of each primer set was verified by melting curve analysis and gel electrophoresis. The amounts of cDNA transcripts were normalized to ribosomal protein S9 (RPS9) cDNA. Expression of RPS9 also was compared to β-actin and GAPDH expression to verify its stability over treatment conditions. The relative expression of each gene was determined using the 2^−ΔΔCt^ method [59]. The expression of each gene is relative to the normalized amount of each cDNA transcript in the non-stimulated controls for each experiment.

**Table 1:** Primer sequences for real-time PCR.

Gene (alternate name)	Accesion no.^1^	Strand	Sequence (5′ - 3′)
1α-OHase (CYP27B1)^2^	NM_001192284	Forward Reverse	TGGGACCAGATGTTTGCATTCGC TTCTCAGACTGGTTCCTCATGGCT
24-OHase (CYP24A1)^2^	NM_001191417	Forward Reverse	GAAGACTGGCAGAGGGTCAG CAGCCAAGACCTCGTTGATT
IFN-γ	NM_174086	Forward Reverse	GATTCAAATTCCGGTGGATG GCAGGAGGACCATTACGTTG
IL-17A	NM_001008412	Forward Reverse	TCCATCTCACAGCGAGCACAAG AGCCACCAGACTCAGAAGCAGTA
IL-17F	NM_001192082	Forward Reverse	CTCCCCTTGGGATTACAACA TTCAGGGTCCTGTCTTCCTG
iNOS^2^	NM_001076799	Forward Reverse	GATCCAGTGGTCGAACCTGC CAGTGATGGCCGACCTGATG
RANTES (CCL5)^2^	NM_175827	Forward Reverse	CACCCACGTCCAGGAGTATT CTCGCACCCACTTCTTCTCT
RPS9^2^	NM_001101152	Forward Reverse	GTGAGGTCTGGAGGGTCAAA GGGCATTACCTTCGAACAGA
VDR	NM_001167932	Forward Reverse	AGCCACCGGCTTCCATTTCA AACAGCGCCTTCCGCTTCAT

1 Accesion numbers for mRNA sequences from NCBI database.

2 Primer sequences have been published previously [20].

### Measurement of nitric oxide production

Production of nitric oxide by PBMCs was determined by measurement of nitrite in the culture supernatant by using the Griess assay as previously described [20]. Supernatants (100 µL) from PBMC cultures were added to an equal volume of Griess reagent [0.5% sulfanilamide, 2.5% phosphoric acid, and 0.05% *N*-(1-naphthyl) ethylenediamine dihydrochloride; Sigma-Aldrich] in a 96-well clear bottom plate. Absorbance at 550 nm in each well was measured using a FlexStation 3 plate reader (Molecular Devices, Sunnyvale, CA). Absorbance values were converted to micromoles per liter using a standard curve that was generated by addition of 0 to 100 µ*M* sodium nitrite to fresh culture media.

### Measurement of IFN-γ production

The concentration of IFN-γ in PBMC culture supernatants was determined by an ELISA using the Endogen Bovine IFNγ Screening Set (Pierce Biotechnology, Rockford IL) according to the manufacturers instructions. The absorbance at 450 nm minus the absorbance at 550 nm was measured with the FlexStation 3 plate reader and the values were converted to picograms per milliliter by using a standard curve.

### Statistical Analysis

Analysis of variance was performed using PROC GLM of SAS (SAS Institute INC., Cary, NC). The model accounted for effects of treatment, cell type, and calf or vaccination status. ΔΔCt values were used in the analyses of gene expression. The average ΔΔCt values ± SE were transformed using the equation 2^−ΔΔCt^. The expression of each gene is presented as the mean fold increase ± SE relative to non-stimulated controls. Multiple comparison tests of the means were made using the Tukey adjustment.

## Results

### *M. bovis* PPD-activation of vitamin D signaling in PBMCs

**Figure 1:**
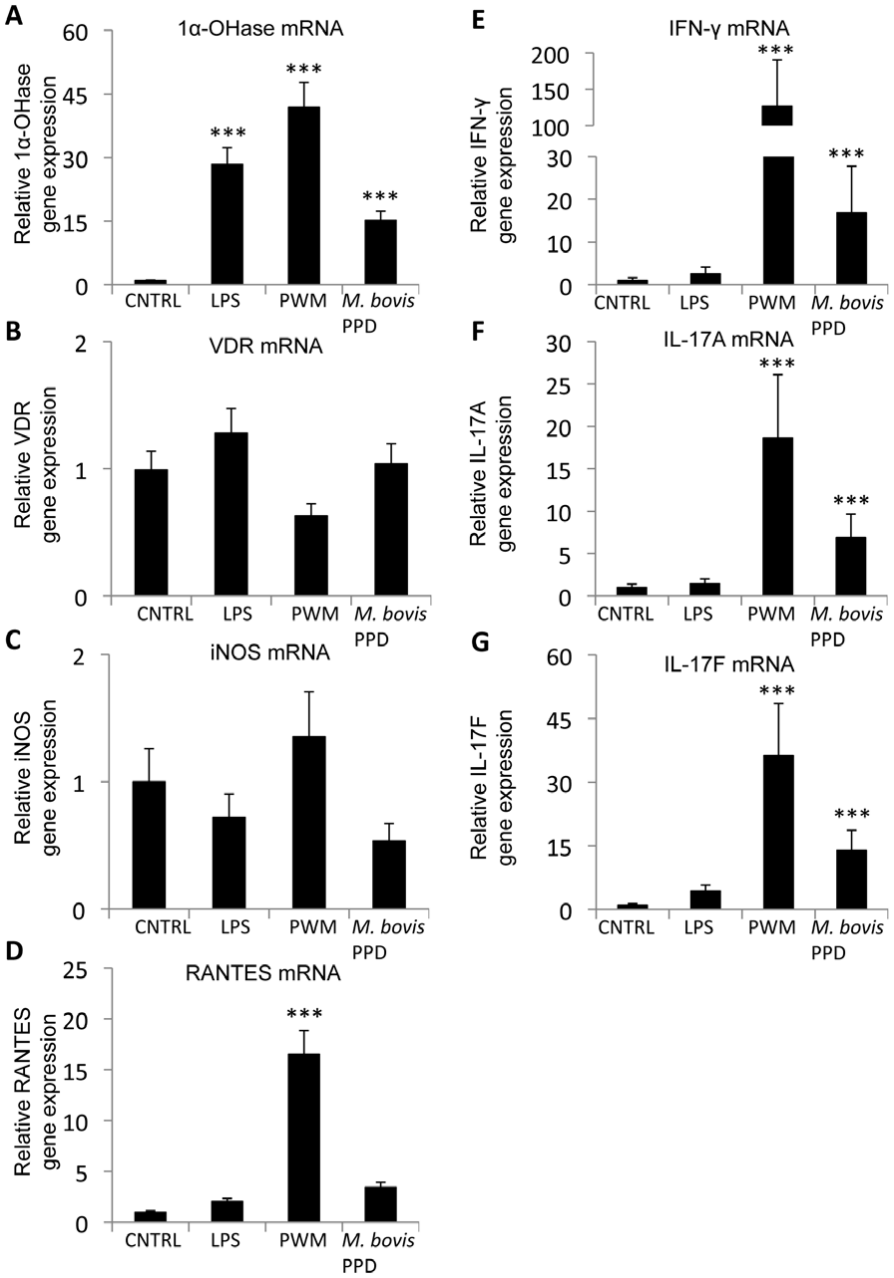
1α-hydroxylase (1α-OHase) gene expression in PBMC cultures. PBMC cultures from eight *M. bovis*-BCG-vaccinated calves were treated with 100 ng/ml LPS, 5 µg/ml PWM, or 10 µg/ml of *M. bovis* PPD or received no treatment (CTRL) as indicated for 24 h. The amount of 1α-OHase (A), VDR (B), iNOS (C), RANTES (D), IFN-γ (E), IL-17A (F), and IL-17F (G) mRNA was determined by quantitative real-time RT-PCR and was normalized to the amount of RPS9 mRNA in each sample. Expression of each gene is relative to non-stimulated cultures. Error bars represent SE. *** Mean is different from non-stimulated PBMC; *P*<0.001.

By stimulating PBMCs from *M. bovis*-BCG-vaccinated calves with LPS, PWM, or *M.* bovis PPD, we found that 1α-OHase gene expression in the PBMCs was upregulated by LPS, PWM, and *M. bovis* PPD stimulation relative to non-stimulated PBMCs (*P*<0.001; Fig. 1A). In contrast, VDR gene expression in the PBMC cultures was not upregulated by any of the stimulants (Fig. 1B). We also measured iNOS, RANTES, IFN-γ, IL-17A, and IL-17F gene expression. Neither iNOS nor RANTES was affected by *M. bovis* PPD or LPS stimulation, but RANTES was upregulated by PWM stimulation (Fig. 1C and D). IFN-γ, IL-17A, and IL-17F were upregulated in PBMCs stimulated with PWM or *M. bovis* PPD, however, they were not affected by LPS stimulation (Fig. 1E–G).

Previously, treatment of *M. bovis* PPD-stimulated PBMCs with exogenous 1,25(OH)_2_D_3_ was found to suppress IFN-γ production [29]. And recently, we showed that conversion of 25(OH)D_3_ to 1,25(OH)_2_D_3_ by 1α-OHase in activated bovine monocytes up-regulated iNOS and RANTES expression [20]. Because 1α-OHase gene expression was upregulated in *M. bovis* PPD-stimulated PBMC, we wanted to determine the effect of 25(OH)D_3_ on gene expression in *M. bovis* PPD-stimulated PBMCs.

**Figure 2:**
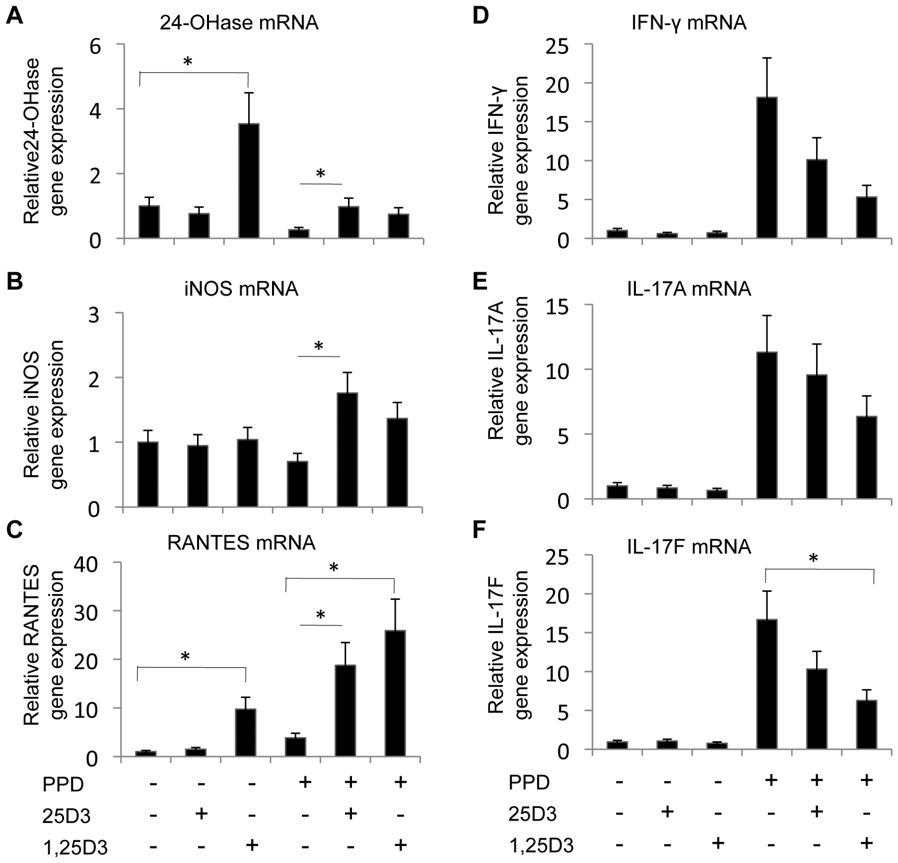
Effects of 1,25(OH)_2_D_3_ and 25(OH)D_3_ on gene expression in PBMC cultures. PBMC cultures from eight *M. bovis*-BCG-vaccinated calves were treated with 0 or 10 µg/ml of *M. bovis* purified protein derivative (PPD), 100 ng/ml of 25(OH)D_3_, and 4 ng/ml 1,25(OH)_2_D_3_ as indicated for 24 h. The amount 24-OHase (A), iNOS (B), RANTES (C), IFN-γ (D), IL17A (E), and IL-17F (F) mRNA was determined by quantitative real-time RT-PCR. Each gene was normalized to the amount of RPS9 mRNA in each sample. Expression of each gene is relative the expression of that gene in non-stimulated cultures. Error bars represent SE. * Means are different; *P*<0.05.

Addition of 100 ng/mL 25(OH)D_3_, a physiological concentration [54], to resting PBMCs did not affect expression of any of the genes tested (Fig. 2). Addition of 4 ng/mL 1,25(OH)_2_D_3_, a concentration 2 to 3 orders of magnitude greater than normal serum 1,25(OH)_2_D_3_, did upregulate 24-OHase and RANTES gene expression in resting PBMCs (*P*<0.05; Fig. 2A and C). Stimulation of PBMCs with *M. bovis* PPD suppressed 24-OHase gene expression (*P*<0.05; Fig. 2A), which is consistent with LPS stimulation of bovine monocytes [20]. However, addition of either 25(OH)D_3_ or 1,25(OH)_2_D_3_ to stimulated PBMCs increased 24-OHase gene expression relative to PBMCs that were stimulated with *M. bovis* PPD alone. Similarly, iNOS and RANTES were upregulated in *M. bovis* PPD-stimulated PBMCs treated with 25(OH)D_3_ or 1,25(OH)_2_D_3_ compared to PBMCs that were stimulated with *M. bovis* PPD alone (*P*<0.05; Fig. 2B and C). IL-17F gene expression was decreased in stimulated PBMCs that were treated with 1,25(OH)_2_D_3_ (*P*<0.05; Fig. 2F). IFN-γ, IL-17A, and IL-17F gene expression was decreased by 25(OH)D_3_ treatment, but the decrease was not statistically significant (*P*>0.05; Fig. 2D–F).

**Figure 3:**
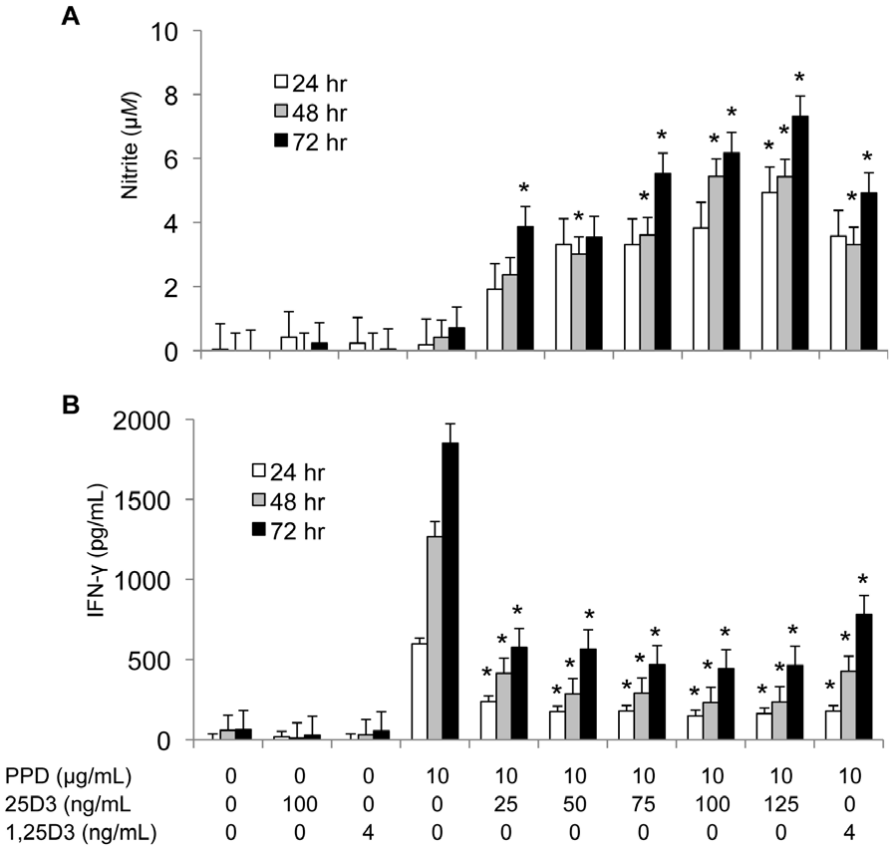
Effects of 1,25(OH)_2_D_3_ and 25(OH)D_3_ on nitrite oxide and IFN-γ production. PBMCs from seven *M. bovis*-BCG-vaccinated calves were cultured at a concentration of 1×10^6^ cells/mL with 0 or 10 µg/ml of *M. bovis* purified protein derivative (PPD), 25 to 125 ng/ml of 25(OH)D_3_, or 4 ng/ml 1,25(OH)_2_D_3_ as indicated for 24, 48, and 72 h. A) The nitrite concentration in the culture supernatants was measured by using the Griess assay and used as an indicator of nitric oxide production by the PBMCs. B) The concentration of IFN-γ in the culture supernatants was measured by an ELISA specific for bovine IFN-γ. Data represents the average concentration of nitrite or IFN-γ in culture supernatants harvested at 24, 48, and 72 h. Error bars represent SE. * Mean is different (*P*<0.05) from PBMC cultures treated with *M. bovis* PPD alone for the corresponding time point.

In addition to gene expression, we measured nitric oxide and IFN-γ production by *M. bovis* PPD-stimulated PBMCs treated with graded doses of 25(OH)D_3_ form 0 to 125 ng/mL (Fig. 3). Like iNOS gene expression, 100 ng/mL 25(OH)D_3_ increased nitric oxide production, as measured by nitrite in the culture supernatant, after 48 and 72 h in culture (Fig. 3A). Furthermore, treatment with 25 to 125 ng/mL 25(OH)D_3_ increased nitric oxide production by the stimulated PBMCs in a dose dependent manner. Addition of 25(OH)D_3_ suppressed IFN-γ production in *M. bovis* PPD-stimulated cultures after 24, 48, and 72 h in culture (*P*<0.05; Fig. 3B), but the effect of 25(OH)D_3_ did not occur in linear fashion.

### Cell type-specific expression of 1α-OHase and VDR

**Figure 4:**
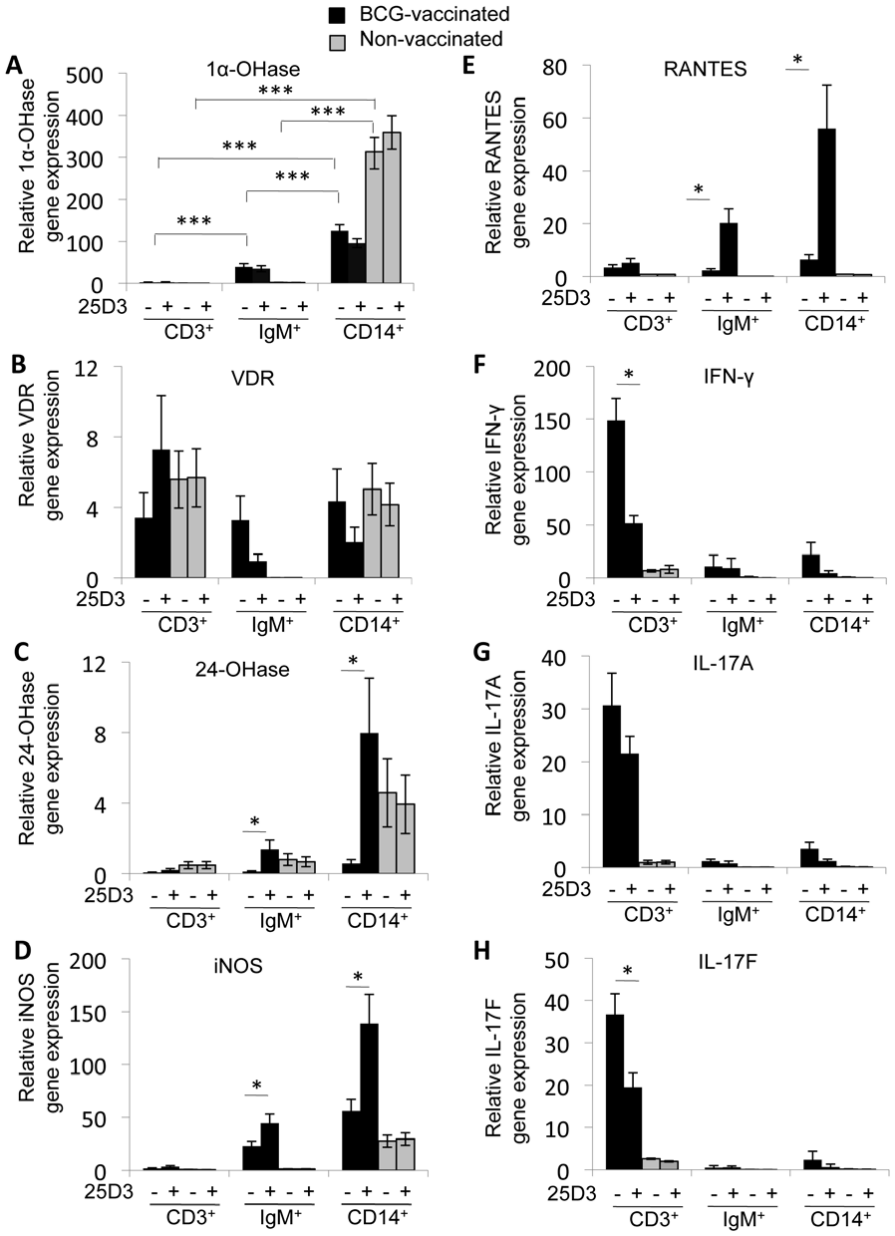
Cell-type specific regulation of vitamin D signaling. PBMCs from seven *M. bovis*-BCG-vaccinated calves (black bars) and four non-vaccinated calves (grey bars) were treated with 10 µg/ml of *M. bovis* PPD and 0 or 100 ng/ml 25(OH)D_3_ as indicated for 24 h. After treatment, PBMCs were sorted by FACS according to CD3/4/8/γδTCR (CD3; T cell), IgM (B cell), and CD14 (monocyte) expression on the cell surface. The amount of 1α-OHase (A), VDR (B), 24-OHase (C), iNOS (D), RANTES (E), IFN-γ (F), IL-17A (G), and IL-17F (H) mRNA in the sorted cells was determined by quantitative real-time RT-PCR. The amount of mRNA for each gene was normalized to the amount of RPS9 mRNA in each sample. Expression of each gene in each cell type is relative to non-stimulated, non-sorted PBMCs. Error bars represent SE. * *P*<0.05, *** *P*<0.001, Means are different.

Several cell types have been reported to express 1α-OHase, including activated monocytes, T cells, and B cells [41], [43], [44]. We sorted PBMCs that had been stimulated with *M. bovis* PPD from BCG-vaccinated animals according to surface expression of CD3, IgM, and CD14 by using FACS (Fig. 4) to determine what populations of cells in PBMCs were expressing 1α-OHase upon activation. By sorting the stimulated PBMCs, we found that 1α-OHase was predominantly expressed in the CD14^+^ population of cells (*P*<0.001; Fig. 4A). 1α-OHase expression was also induced in IgM^+^ cells from vaccinated calves (*P*<0.001). Relative to 1α-OHase expression in non-stimulated, non-sorted PBMCs, the expression of 1α-OHase did not increase in the CD3^+^ cells isolated from the stimulated PBMC cultures (Fig. 4A). Unlike 1α-OHase, VDR gene expression did not differ significantly between cell types in PBMC cultures from vaccinated calves, but IgM^+^ cells from 25(OH)D_3_ treated cultures did have somewhat lower VDR expression (Fig. 4B).

### Cell type-specific effects of 25(OH)D_3_ on gene expression

We also compared gene expression in cells from *M. bovis* PPD-stimulated PBMCs that were treated with 100 ng/ml 25(OH)D_3_ with cells from *M. bovis* PPD-stimulated PBMCs that were not treated with 25(OH)D_3_. Treatment with 25(OH)D_3_ increased 24-OHase, iNOS, and RANTES gene expression in both CD14^+^ cells and IgM^+^ cells from the BCG-vaccinated calves (*P*<0.05; Fig. 4C–E). In contrast, 25(OH)D_3_ treatment decreased expression of IFN-γ by over 60% and IL-17F by nearly 50% in the CD3^+^ cells from the BCG-vaccinated calves (*P*<0.05; Fig. 4F and H). IL-17A expression in the CD3^+^ cells was also down-regulated by 25(OH)D_3_ treatment, but to a lesser extent (*P*>0.05; Fig. 4G).

### Comparison of responses between BCG-vaccinated and non-vaccinated animals

Finally, we compared changes in gene expression caused by *M. bovis* PPD stimulation and 25(OH)D_3_ in cells from non-vaccinated animals to the changes observed in cells from BCG-vaccinated animals. In PBMCs from non-vaccinated animals, 1α-OHase was induced in CD14^+^ cells by *M. bovis* PPD stimulation like in CD14^+^ cells from BCG-vaccinated animals (Fig. 4A). Unlike the PBMCs from BCG-vaccinated calves, VDR was not detected in IgM^+^ cells from the non-vaccinated animals (Fig. 4B). VDR expression in CD3^+^ and CD14^+^ cells was similar between vaccinated and non-vaccinated calves. Neither 24-OHase, iNOS, or RANTES expression was affected by 25(OH)D_3_ in CD14^+^ cells and IgM^+^ cells from the non-vaccinated animals like it was in the BCG-vaccinated animals (Fig. 4C–E). Finally, IFN-γ, IL-17A, and IL-17F were not induced by *M. bovis* PPD stimulation in the CD3^+^ cells from non-vaccinated animals as they were in CD3^+^ cells from BCG-vaccinated animals (Fig. 4F–H).

## Discussion

For over two decades now, 1,25(OH)_2_D_3_ has been known as an important regulator of adaptive immunity, suppressing lymphocyte proliferation and IFN-γ production [28], [60]. The implications of 1,25(OH)_2_D_3_ on adaptive immunity are further realized in animal models of T cell-mediated autoimmunity as 1,25(OH)_2_D_3_ inhibits disease progression [32], [34], [61], [62]. Recently, 1,25(OH)_2_D_3_ also was found to be an important regulator of innate immunity by enhancing antimicrobial properties of macrophages [16], [18]. Vitamin D-mediated immune responses, however, do not correlate with the circulating concentration of 1,25(OH)_2_D_3_ [38], [39]. Therefore, local synthesis of 1,25(OH)_2_D_3_ is a critical factor in regulating both innate and adaptive immunity. Previously, induction of 1α-OHase expression in macrophages was shown to occur upon activation by TLR 2/1 or TLR 4 signaling and enable them to convert 25(OH)D_3_ to 1,25(OH)_2_D_3_ [20], [41], [63]. Synthesis of 1,25(OH)_2_D_3_ in macrophages, in turn, enhanced their innate antimicrobial properties in an intracrine fashion [48]. In this study, we give evidence that endogenous synthesis of 1,25(OH)_2_D_3_ also occurs in antigen-stimulated PBMC cultures and regulates key aspects of adaptive immunity.

**Figure 5:**
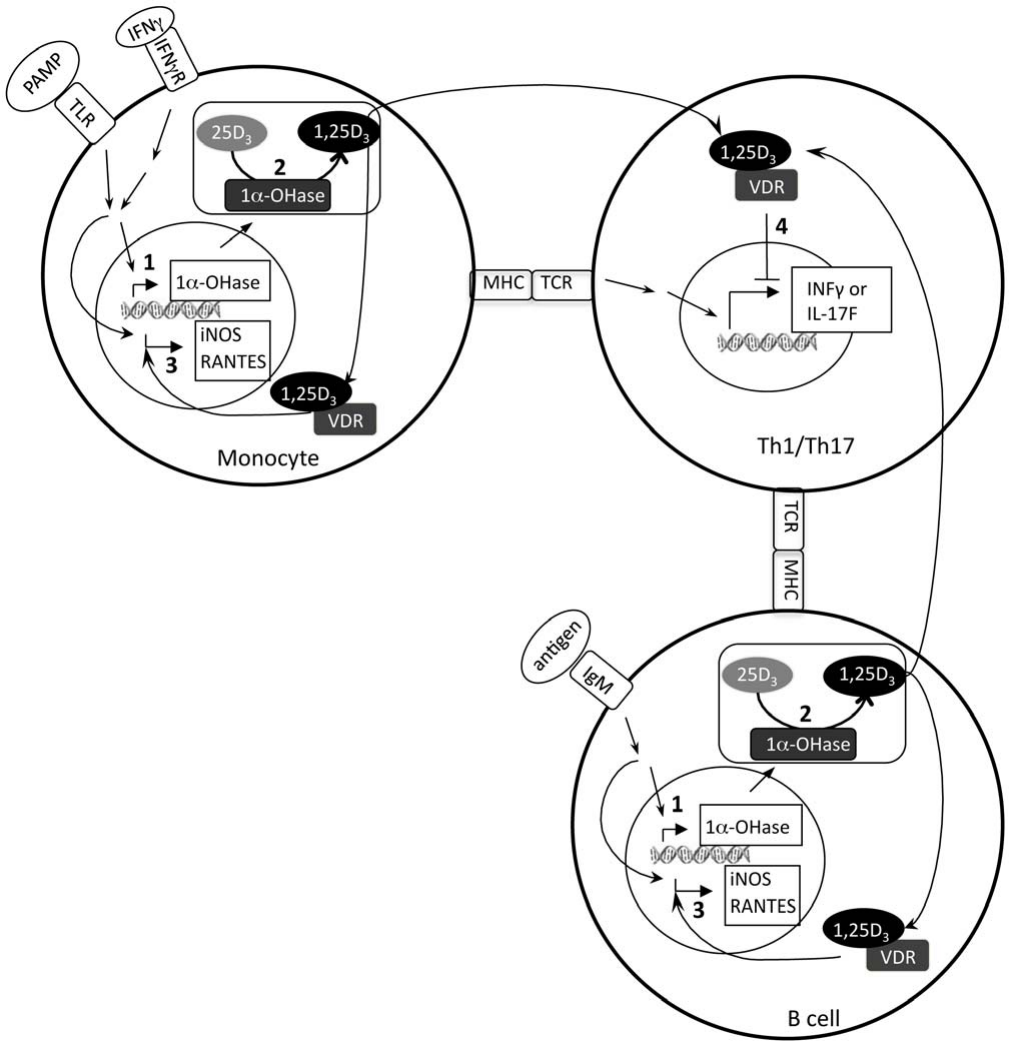
Regulation of T cells through a paracrine mechanism of vitamin D signaling. 1) 1α-hydroxylase (1α-OHase) gene expression is induced in monocytes by recognition of pathogen associated molecular patterns (PAMP) by toll-like receptors (TLR) and interferon-γ (IFN-γ) from Th1 cells, and in B cells by antigen recognition by the B cell receptor (IgM) along with co-stimulation by antigen-specific T cells. 2) 25-hydroxyvitamin D_3_ (25D_3_) is converted to 1,25-dihydroxyvitamin D_3_ (1,25D_3_) by 1α-OHase. 3) 1,25(OH)_2_D_3_ produced in monocytes and B cells activates the vitamin D receptor (VDR) and enhances iNOS and RANTES expression in monocytes and B cells. 4) 1,25(OH)_2_D_3_ secreted from monocytes and B cells suppresses IFN-γ and interleukin-17F (IL-17F) expression in T cells. Direct suppression of IFN-γ and IL-17F expression by the 1,25(OH)_2_D_3_, however, is not certain. Instead, 1,25(OH)_2_D_3_ may influence T cell development, suppress proliferation, or sensitize them to apoptosis.

In this study we found that 1α-OHase gene expression was induced in CD14^+^ cells (monocytes/macrophages) and IgM^+^ (B cells), but not in CD3^+^ (T cells) cells in *M. bovis* PPD-stimulated PBMC cultures. Furthermore, treatment of *M. bovis* PPD-stimulated PBMC cultures with 25(OH)D_3_ enhanced iNOS and RANTES expression in monocytes and B cells and suppressed antigen-specific IFN-γ and IL-17F responses in T cells. Based on this evidence, we hypothesize that 1,25(OH)_2_D_3_ was produced in monocytes and B cells acted on monocytes and B cells in an intracrine fashion to upregulate iNOS and RANTES expression and on T cells in a paracrine fashion to suppress *M. bovis* PPD-specific IFN-γ and IL-17F responses (Fig. 5).

*M. bovis* PPD is a crude extract and as such likely contains antigens that activate both the innate and adaptive immune systems. Therefore, in PBMC cultures innate antigen presenting cells (e.g., monocytes) recognize TLR ligands, such as lipoproteins, become activated and then express 1α-OHase. The APCs also internalize protein from the *M. bovis* PPD, process it and then present it on their surface as peptide associated with MHC. Activation of T cells specific for *M. bovis* PPD is then caused by the interaction of the specific T cell receptor (TCR) with its cognate MHC/antigen. Likewise, B cells recognize antigen through IgM on their surfaces, and along with co-stimulation from T cells, become activated and express 1α-OHase. We suggest that production of 1,25(OH)_2_D_3_ by 1α-OHase in activated monocytes and B cells can alter the IFN-γ and IL-17F responses that are the result of the TCR/MHC/antigen interaction between T cells and APCs.

There are multiple possibilities as to how 1,25(OH)_2_D_3_ suppressed IFN-γ and IL-17F gene expression in T cells. VDR expression in the T cells was similar to that in monocytes in this study and purified T cells do respond to 1,25(OH)_2_D_3_ [25], . Also, T cell VDR expression is required for 1,25(OH)_2_D_3_-mediated inhibition of experimental autoimmune encephalomyelitis in mice [35]. Therefore, the T cells in the PBMC cultures likely had the ability to respond to 1,25(OH)_2_D_3_ secreted from the monocytes and B cells. Consequently, activation of the VDR in T cells could have directly suppressed IFN-γ and IL-17F expression. However, 1,25(OH)_2_D_3_ failed to suppress IFN-γ production in fully differentiated Th1 cells [61]; so, 1,25(OH)_2_D_3_ may have regulated genes in T cells that influenced T cell differentiation or sensitized them to apoptosis. Alternatively, up-regulation of nitric oxide production by 1,25(OH)_2_D_3_ in monocytes and B cells could have induced apoptosis in the surrounding T cells and resulted in suppressed IFN-γ and IL-17F expression. A combination of several mechanisms also is possible and we have not ruled out the possibility that T cells are able to synthesize their own 1,25(OH)_2_D_3_. Therefore, further experiments are needed to determine if regulation of T cell responses by 1,25(OH)_2_D_3_ strictly depends on synthesis of 1,25(OH)_2_D_3_ in monocytes or B cells and how 1,25(OH)_2_D_3_ is regulating T cell responses.

In any case, treatment of antigen-stimulated PBMCs with 25(OH)D_3_ suppressed antigen-specific IFN-γ and IL-17F expression in T cells, which indicates that synthesis of 1,25(OH)_2_D_3_ by immune cells has significant implications in regulating adaptive immunity. IFN-γ is a potent activator of macrophages and is mainly produced by Th1 cells [65]. Th1-mediated responses are critical in the defense against intracellular infections, such as tuberculosis [66], [67]. IL-17A and IL-17F are produced by Th17 cells and play major roles in neutrophil recruitment and protection against intracellular and extracellular bacterial infections [68], [69], [70]. Self reactive Th1 and Th17 cells, however, are involved in the development of autoimmune disorders [71] and inhibition of animal models of autoimmunity by 1,25(OH)_2_D_3_ is thought to occur, in part, by suppression of self-reactive Th1 and Th17 cells [72]. Although suppression of Th1 and Th17 responses to bacterial antigens by 1,25(OH)_2_D_3_ would seem to attenuate the immune response against bacterial infections, keep in mind that 1,25(OH)_2_D_3_ also enhances the antimicrobial activity of macrophages [41]. So overall, production of 1,25(OH)_2_D_3_ by immune cells serves to limit inflammation caused by Th1 and Th17 effector cells, but ultimately improves defense against bacterial infections by boosting the innate antimicrobial response.

In addition to suppression of T cell responses by 1,25(OH)_2_D_3_ synthesis in PBMC cultures, treatment of PBMCs with 25(OH)D_3_ upregulated antigen-specific B cell iNOS and RANTES expression. We had previously shown that monocyte iNOS and RANTES expression depends on availability of 25(OH)D_3_ [20], but not B cell iNOS and RANTES. Nitric oxide produced by iNOS in macrophages is considered to be an antimicrobial molecule. However, nitric oxide produced by the monocytes and B cells may suppress proliferation of T cells [73]. So, as mentioned above, 1,25(OH)_2_D_3_ may suppress T cell responses in part by enhancing B cell and monocyte iNOS expression. RANTES is a chemokine originally found to be expressed by T cells [74], but also has been found to be expressed by alveolar macrophages in cattle [75]. We speculate that upregulation of RANTES in monocytes and B cells by 1,25(OH)_2_D_3_ would enhance recruitment of immune cells to the site of inflammation, but the implications of 1,25(OH)_2_D_3_-upregulation of RANTES in monocytes and B cells will need to be investigated.

Finally, the ability of 1α-OHase in monocytes and B cells to synthesize 1,25(OH)_2_D_3_, and subsequently regulate 1,25(OH)_2_D_3_-mediated immune responses, depends on the availability of 25(OH)D_3_. The circulating concentration of 25(OH)D is primarily regulated by dietary intake of vitamin D_3_ and sun exposure [76]. Current recommendations for vitamin D in humans and cattle target a circulating concentration of 25(OH)D of 20 to 50 ng/ml [55], [77]. However, 25(OH)D concentrations above 30 ng/ml may be necessary for optimal immune function [1]. In addition, vitamin D insufficiency (serum 25(OH)D below 30 ng/ml) and even deficiency (serum 25(OH)D below 20 ng/ml) is widespread, indicating that current recommendations for vitamin D_3_ intake may be inadequate [78], [79]. Previously, and here, we have shown that 1,25(OH)_2_D_3_-regulated innate immune responses increase linearly from 0 to 125 ng/ml of 25(OH)D_3_ [20]. This observation leads to the question of what concentration is necessary for optimal immune functionality if below 30 ng/ml is insufficient? Based on the requirement of 25(OH)D_3_ by the immune system for signaling mechanisms and evidence from epidemiological studies, vitamin D requirements need to be re-evaluated to ensure proper immune function.
